# Characterization of Amylolysin, a Novel Lantibiotic from *Bacillus amyloliquefaciens* GA1

**DOI:** 10.1371/journal.pone.0083037

**Published:** 2013-12-09

**Authors:** Anthony Arguelles Arias, Marc Ongena, Bart Devreese, Mohammed Terrak, Bernard Joris, Patrick Fickers

**Affiliations:** 1 Centre d'Ingénierie des Protéines, Bacterial Physiology and Genetics, Université de Liège, Liège, Belgium; 2 Centre Wallon de Biologie Industrielle, Unité de Bioindustrie, Faculté de Sciences Agronomique de Gembloux, Gembloux, Belgium,; 3 Laboratorium voor Eiwitbiochemie en Eiwitengineering, Universiteit-Gent, Gent, Belgium; 4 Unité de Biotechnologies et Bioprocédés, Université Libre de Bruxelles, Brussels, Belgium; Institut Pasteur Paris, France

## Abstract

**Background:**

Lantibiotics are heat-stable peptides characterized by the presence of thioether amino acid lanthionine and methyllanthionine. They are capable to inhibit the growth of Gram-positive bacteria, including *Listeria monocytogenes, Staphylococcus aureus* or *Bacillus cereus*, the causative agents of food-borne diseases or nosocomial infections. Lantibiotic biosynthetic machinery is encoded by gene cluster composed by a structural gene that codes for a pre-lantibiotic peptide and other genes involved in pre-lantibiotic modifications, regulation, export and immunity.

**Methodology/Findings:**

*Bacillus amyloliquefaciens* GA1 was found to produce an antimicrobial peptide, named amylolysin, active on an array of Gram-positive bacteria, including methicillin resistant *S. aureus*. Genome characterization led to the identification of a putative lantibiotic gene cluster that comprises a structural gene (*amlA*) and genes involved in modification (*amlM*), transport (*amlT*), regulation (*amlKR*) and immunity (*amlFE*). Disruption of *amlA* led to loss of biological activity, confirming thus that the identified gene cluster is related to amylolysin synthesis. MALDI-TOF and LC-MS analysis on purified amylolysin demonstrated that this latter corresponds to a novel lantibiotic not described to date. The ability of amylolysin to interact *in* *vitro* with the lipid II, the carrier of peptidoglycan monomers across the cytoplasmic membrane and the presence of a unique modification gene suggest that the identified peptide belongs to the group B lantibiotic. Amylolysin immunity seems to be driven by only two AmlF and AmlE proteins, which is uncommon within the *Bacillus* genus.

**Conclusion/Significance:**

Apart from mersacidin produced by *Bacillus amyloliquefaciens* strains Y2 and HIL Y-85,544728, reports on the synthesis of type B-lantibiotic in this species are scarce. This study reports on a genetic and structural characterization of another representative of the type B lantibiotic in *B. amyloliquefaciens*.

## Introduction

Bacteriocins are antimicrobial peptides ribosomally synthesized capable to inhibit the growth of Gram-positive bacteria, including *Listeria monocytogenes, Staphylococcus aureus* or *Bacillus cereus*, the causative agents of food-borne diseases or nosocomial infections [1,2]. The class I bacteriocins, the so-called lantibiotics, are heat stable post-translationally modified peptides that contain multiple thioether amino acids lanthionine (Lan) and methyllanthionine (Melan)[3]. These latter are respectively enzymatically synthesized from a cysteine thiol and the dehydrated dehydroalanine (Dha) or didehydrobutyrine (Dhb) amino acids [4].

 Lantibiotics could be subdivided into two main subgroups: type-A lantibiotics that exhibit a linear secondary structure and are positively charged at neutral pH. They are modified by two distinct LanB and LanC enzymes and processed by a LanP protease. Type-B lantibiotics, conversely, exhibit a globular structure and are non-charged or slightly negatively charged at neutral pH. They are modified by a single modification LanM enzyme and processed by a LanT ABC transporter with N-terminal-associated protease activity [5]. Type-B subgroup also includes the so-called two-component lantibiotics consisting of two synergistically acting peptides that are modified by a single LanM-type enzyme [6]. Recently, a third Type-C subgroup has been reported. It corresponds to peptides, such as SapT and SapB from *Streptomyces tendae*, that present mainly a morphogenetic function rather than an antimicrobial activity. Beside this, lantibiotic could also be distinguished based on their biological mode of action. Some, such as mersacidin, bind to lipid II and thereby inhibit peptidoglycan cell wall synthesis in sensitive Gram-positive bacteria [7] while others, such as Pep5, form pores in cytoplasmic membrane that lead to cell leakage and finally to cell death [8,9]. A third group of compounds is formed by lantibiotic that possess a dual mode of action, i.e. inhibition of the peptidoglycan biosynthesis and pore forming. Both mode of action could be ensured by a single peptide (such as nisin) or by two distinct peptides in two-component lantibiotic, such as lacticin 3147 [10]. 

 Lantibiotic peptides are gene encoded and their structural genetic determinants are found in biosynthetic gene clusters. They are synthesized as inactive prepeptides, with a N-terminal leader sequence separated from the mature lantibiotic [3]. In type A lantibiotics, many of the leader peptides end with a PQ or PR sequence and share a conserved F(N/D)LD motif in their core sequence. On the other hand, leader peptides of type-B lantibiotics end with G(G/A/S) sequence [6]. They are not processed by a LanP protease such as in type-A lantibiotic but by the N-terminal intracellular domain of LanT protein [11]. In addition, all lantibiotic gene clusters encode a set of immunity proteins that protect the producer strain against the biological effect of the synthesized lantibiotic. Depending on the lantibiotic considered, these immunity proteins could be LanI, LanF, LanE or LanG [12]. Regulation of lantibiotics biosynthesis is mediated by regulatory LanR and LanK proteins that constitute a two-component signal transduction system [13].

 Despite the majority of the lantibiotics described so far are from lactic bacteria, *Bacillus* represents an alternative genus to investigate for antimicrobial peptides because it includes many industrial species and has a history of safe use in the food industry [14]. There are few reports on lantibiotic synthesis in *B. amyloliquefaciens*. Production of mersacidin, a type-B lantibiotic, has been reported in *B. amyloliquefaciens* subsp *plantarum* B6901-Y2 and *B. amyloliquefaciens* HIL Y-85,54728 (formerly *Bacillus* sp. HIL Y-85,54728) [7,15,16]. The biosynthetic cluster involved in mersacidin biosynthesis is composed of ten genes that span over 12.3 kb in the genome [6]. The biological mode of action of mersacidin is related to its ability to bind to the lipid II and thus to prevent peptidoglycan biosynthesis [7].

Recently, we have identified *Bacillus amyloliquefaciens* GA1 as a producer of a proteinaceous compound with potent antimicrobial activity toward the foodborne pathogen *Listeria monocytogenes* [17]. The failure of structural gene detection for all the genetically described bacteriocins from the *Bacillus* genus strongly suggests that this antimicrobial peptide, named amylolysin, corresponds to a novel bacteriocin not described to date. Prior intensive characterization, amylolysin was purified to test its ability to inhibit the growth of *L. monocytogenes* in poultry meat upon long-term storage [17]. In the present paper, we report on the biochemical characterization of that novel bacteriocin, the nucleotide sequence of the gene cluster involved in its biosynthesis and peculiar features on its inhibition spectrum and structural traits. 

## Results

### Inhibition spectrum

The biological activity of the purified amylolysin was characterized by determining the minimal inhibitory concentration (MIC) for an array of bacterial and fungal indicator strains. As shown in table 1, amylolysin showed an antibacterial spectrum directed toward Gram-positive bacteria. Indeed, no growth inhibition was observed in our experimental conditions neither on both *Basidiomycetous* and *Ascomycetous* yeasts (i.e. *Cryptococcus neoformans* and *Saccharomyces cerevisiae*, respectively), nor on Gram-negative bacterium (i.e. *Escherichia coli* and *Pseudomonas aeruginosa*). By contrast, a significant inhibitory effect was observed for *Enterococcus faecium* with a MIC value of 0.1 µM and for *Enterococcus faecalis* in a lesser extent. Among the *Bacillus* genus, the opportunistic pathogen *B. cereus* that is a common cause of food poisoning was also found very sensitive with an MIC value of 0.2 µM. The growth of *L. monocytogenes*, another major food poisoning bacteria was also found sensitive to amylolysin with an MIC value close 0.5 µM for the three clinical or food isolates tested, confirming thus previous observation [17]. *S. aureus*, including methicillin-resistant isolates (0.4 µM), together with *S. epidermidis* (2.8 µM), which are both opportunistic human pathogens, were also found sensitive to amylolysin. For lactic acid bacteria such as *Weissella* *sp.* and *Lactobacillus plantarum*, only a weak or no growth inhibition effect was observed in our experimental conditions.

**Table 1:** Inhibition spectrum of amylolysin.

**Strains**	**Culture medium,Temperature (°C)**	**MIC (µM)**
*Micrococcus luteus* ATCC 9341	BH, 37	0.7
*Staphylococcus epidermis* ATCC 1228	BH, 37	2.8
*Staphylococcus aureus* ATCC 25923	BH, 37	2.8
*Staphylococcus aureus* ATCC 43300**^*a*^**	BH, 37	0.4
*Staphylococcus aureus* RFB127 **^*b*^**	BH, 37	1.4
*Enterococcus faecalis* ATCC 29212	BH, 37	1.4
*Enterococcus faecalis* RFB129 **^*c*^**	BH, 37	0.7
*Enterococcus faecium* RFB128 **^*b*^**	BH, 37	0.1
*Listeria monocytogenes* LMG 23905	BH, 37	0.4
*Listeria monocytogenes* LMG 21263	BH, 37	0.5
*Listeria monocytogenes* LM2234 **^*c*^**	BH, 37	0.4
*Listeria innocua* ATCC33090	BH, 37	0.7
*Listeria innocua* RFB159**^*c*^**	BH, 37	0.7
*Listeria ivanovii* RFB160	BH, 37	0.8
*Bacillus cereus* RFB125 **^*c*^**	LB, 37	0.2
*Bacillus subtilis* ATCC 6633	LB, 37	1.4
*Bacillus megaterium* RFB124	LB, 37	0.4
*Streptococcus agalactiae* RFB141 **^*c*^**	BH, 37	2.8
*Weissella* *sp* RFB139 **^*b*^**	MRS, 27	2.8
*Lactobacillus plantarum* RFB138 **^*b*^**	MRS, 27	>2.8
*Escherichia coli* RFB149 **^*c*^**	LB, 37	>2.8
*Pseudomonas aeruginosa* RFB148 **^*b*^**	LB, 37	>2.8
*Cryptococcus neoformans* IHEM3969	YPD, 30	>2.8
*Saccharomyces cerevisiae* RFY100	YPD, 30	>2.8

^a^ Methicillin resistant.

^b^ Lab stock.

^c^ Clinical isolates.

LMG and IHEM: http://bccm.belspo.be.

### Amylolysin susceptibility to proteases, heat and pH

Incubation of purified amylolysin with pronase and proteinase K led to a strong decrease of the antimicrobial activity against the indicator strain *Micrococcus luteus* ATCC 9341 (Table **2**). By contrast, no significant reduction of the amylolysin biological activity against the indicator strain was observed upon heat treatments at different temperatures or incubation at various pH (Table **2**). Indeed, incubation of purified amylolysin at 100 °C for 1 hour, led only to a 7% reduction of its biological activity whereas incubation in acidic (pH 2) and alkaline (pH9) condition led to an 11% and 7% antimicrobial activity reduction, respectively. These results were further confirmed by the comparison of the HPLC chromatograms corresponding to treated and non-treated amylolysin samples (data not shown). This demonstrates that amylolysin correspond to a heat and pH stable proteinaceous compound.

**Table 2:** Amylolysin stability.

Factors	Remaining activity (%)
**Protease**	
Control	100
Proteinase K	0
Pronase	20
**PH** ^a^	
2	89
3	96
4	96
5	99
6	96
7	100
8	97
9	93
**Temperature** ^b^	
25°C	100
55°C	98
65°C	99
75°C	99
100°	93

^a^ Values were normalised as a percentage of the value obtained at physiological pH

^b^ Values were normalised as a percentage of the value obtained at 25°C

### Gene cluster sequencing and characterization

**Figure 1:**
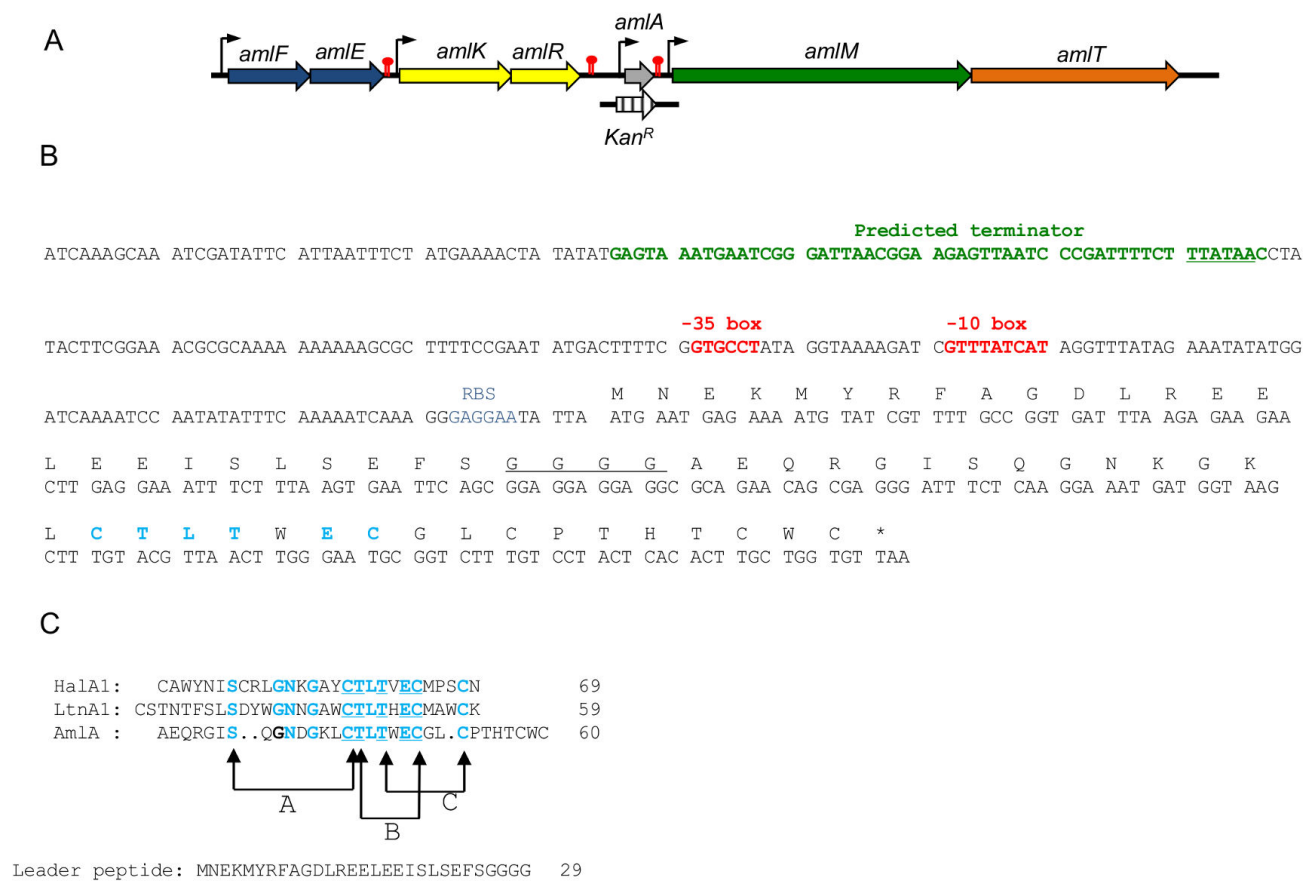
Characterization of the amylolysin gene cluster. (**A**). The gene coding for the amylolysin prepeptide (amlA) is colored in grey. The other genes are marked with the following patterns: immunity (amlEF) in blue, regulation (amlKR) in yellow, modification (amlM) in green and transport in orange. Stripes arrow corresponds to the Kan construct used for *amlA* disruption. Putative promoters and terminators are indicated by vertical arrows and red symbol, respectively. (**B**) Primary structure of *amlA* gene. Predicted -10box and -35box are in red, ribosome binding site (RBS) is in blue and predicted terminator of *amlKR* cluster is in green. The four-glycine stretch is underlined and the highly conserved motif CTLTXEC is in bold blue. (C) Amino acid alignment of AmlA with LanA1 peptides of the two-peptide lantibiotics haloduracin (HalA1, DAB04173) and lacticin 3147 (LtnA1, O87236). Conserved amino acids are in bold blue; those forming the CTLTXEC motif are in bold and underlined. The thioether bridging patterns represents that of HalA1.

*In silico* analysis of the 461.5 kb fragment of the *B. amyloliquefaciens* GA1 chromosome, obtained by partial shotgun sequencing [18], led to the identification of a 800 bp fragment encoding an amino acid sequence that exhibits 38 % and 33 % identity with the lantibiotic modification enzyme from *Bacillus licheniformis* ATCC 14580 (Genbank identifier (GI): 304557386) and *Bacillus halodurans* C-125 (GI: 15613018), respectively. Further characterization of this locus led to the identification of different genes homologous to genes involved in lantibiotic biosynthesis. Indeed, from a 5.5 kb fragment obtained by inverse polymerase chain reaction (IPCR) [19], four complete ORFs were identified (Figure **1A**). Of these, the so-called *amlA* codes for a polypeptide of 60 residues that presents 35 % and 40 % of identity with the MrsA mersacidin peptide from *B. amyloliquefaciens* HIL Y-85,54728 and *Bacillus pseudomycoides* DSM 12442, respectively. A detailed analysis of AmlA sequence revealed the presence of a GG sequence corresponding to the signal peptidase cleavage site of type-B lantibiotics (Figure **1B**). The GxxxxTx(S/T)x(D/E)C(3-10)xC motif present in all mersacidin and lacticin 481 like peptides was also observed together with the CTxTxEC amino acid sequence known as essential for interactions with the peptidoglycan biosynthesis precursor lipid II [20,21]. Located downstream of *amlA*, *amlM* encodes a putative 908 amino acids protein that exhibits 30 % of identity with the lantibiotic modifying enzyme LchM1 from *Bacillus licheniformis* ATCC 14580. Multiple sequence alignment of AmlM N-terminal domain (residues 64 to 364) with other homologous modification enzymes highlights the presence of the conserved motifs previously described for this type of enzymes (Figure **S1**) [6,22]. 

Beside this*, amlK* and *amlR*, located upstream of *amlA* were found to encode proteins that present strong similarities with two-component regulatory proteins. AmlK shows 32 % of identity with C-terminal cytoplasmic domain of the *Bacillus subtilis* 168 histidine kinase ComP that acts as a membrane sensor of environmental signals. In this conserved domain, His^30^ and Asp^161^ are the autophosphorylated residue and the catalytic amino acid conserved in NisR, SpaR and ComP histidine kinases, respectively [23,24]. In addition, the conserved glycine rich stretch was found between amino acid 163 and 203 [13,24] (data not shown). AmlR exhibits a 46 % of identity with transcriptional regulators belonging to LuxR family (GI: 251795068). Indeed, in its N-terminal sequence, the characteristic K^3^ILxxDD^9^ and L^49^xxLD^53^ motifs (including catalytic residues Asp^9^ and Asp^53^) and the lysine residue (Lys^153^) of response regulators are found. 

In the nucleic acid fragment obtained by IPCR, the amylolysin immunity and transport genes are missing. To identify these later, a BlastX search through prokaryotic protein databases was performed by using the 5.5 kb fragment as the query sequence. For *amlK, amlR* and *amlM*, a high identity (100% on nucleotide level) was found with sequences from *Bacillus amyloliquefaciens* IT45 annotated as sensor histidine kinase (GI: 363725376), LuxR family transcriptional regulator (GI: 363725375) and putative LanM like protein (GI: 363725373), respectively. To obtain the missing sequence of the amylolysin biosynthetic gene cluster, primer walking was performed using *B. amyloliquefaciens* IT45 genome sequence as a template (GI: 423191475). This led to the identification of a 1.9 and 2.1 kb fragment upstream and downstream of *amlK* and *amlA*, respectively (Figure **1**). Of these, three ORFs, designated as *amlT*, *amlF* and *amlE*, were identified and the deduced amino acid sequences are identical to those present in the *B. amyloliquefaciens* IT45 sequence and annotated as ABC transporter-like protein (GI: 363725372), efflux ABC transporter ATP-binding protein (GI: 363725380) and hypothetical protein KSO14349 (GI: 363725379), respectively. A search for specific motifs in these three amino acid sequences highlights the following similarities. The N-terminal part of AmlT belongs to C39 bacteriocin-processing peptidase superfamily [11]. More precisely, Cys^19^, His^97^ and Asp^113^ were identified as the putative catalytic amino acids conserved in these peptidases (data not shown) [25]. Beside this, the C-terminal part of AmlT sequence was found to exhibit similarities with ABC transporter superfamily, suggesting its role in amylolysin transport (data not shown). AmlE exhibits 36% identity with the membrane-bound part of a lantibiotic ABC transporter (GI: 260687109) from *Clostridium difficile* R20291, whereas AmlF shows 44% identity with MrsF (GI: 385266873) from *Bacillus* sp. 5B6, involved in lantibiotic self-protection. This latter contains a conserved domain of ABC transporter and belongs to the P-loop NTPase superfamily (data not shown) [26]. Therefore, it is likely that AmlE and AmlF could be involved in immunity mechanism with AmlF being the ATP-binding subunit and AmlE the efflux protein of an ABC transporter. The different *aml* gene sequences were deposited at GenBank under the accession number KC415250.1 (GI:448918122). 

### Disruption of *amlA* gene

**Figure 2:**
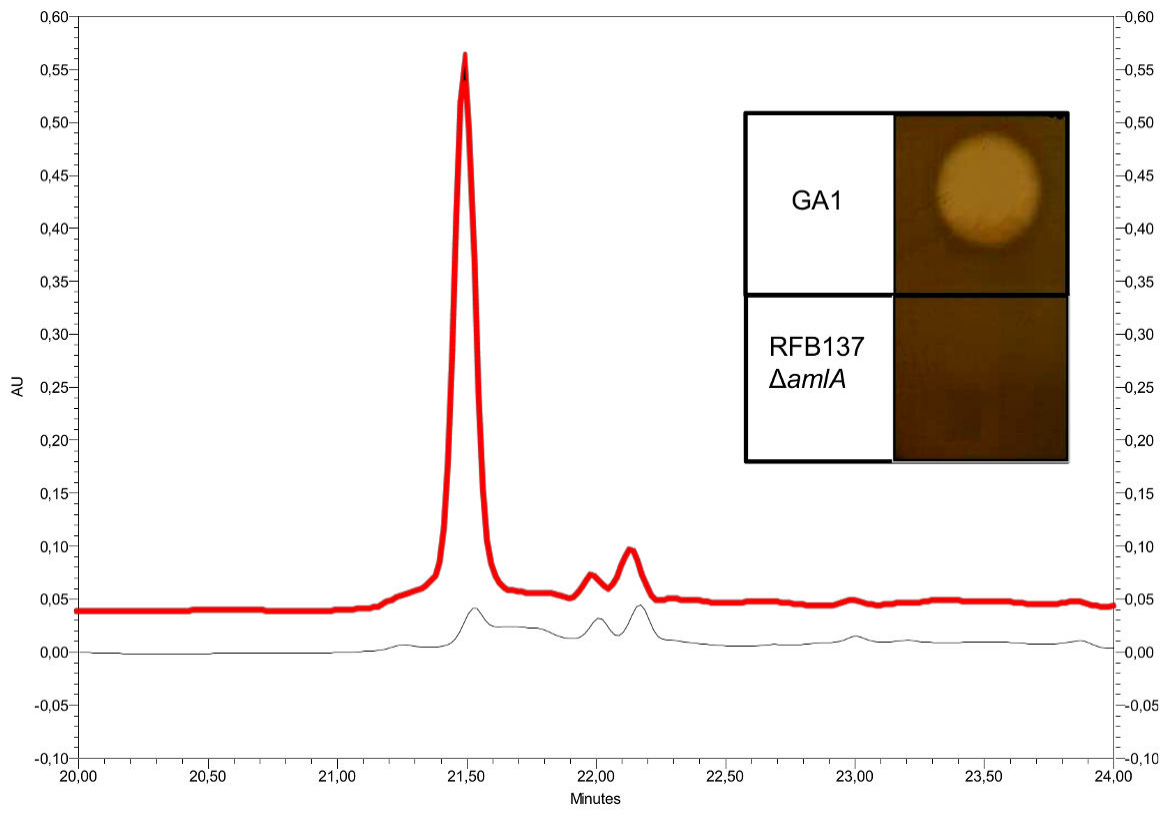
Characterization of the RFB137 mutant. The RP-HPLC chromatogram of concentrated supernatant of GA1 is in red and that of the *ΔamlA* RFB137 mutant is in black. Insert: Antimicrobial activity of concentrated supernatant (10 µl) from GA1 and RFB137 strains on LB agar plates seeded with *M. luteus* ATCC 9341.

In order to correlate the antimicrobial activity of amylolysin to the putative lantibiotic gene cluster, the structural *amlA* gene was disrupted by insertion of a kanamycin resistance gene into *amlA* by double homologous recombination (Figure **1A**). Culture supernatant of the resulting RFB137 insertion mutant (Table **S1**) was characterized by a loss of antibacterial activity against *M. luteus* ATCC 9341 compared to that of the wild-type strain (Figure **2** **, insert**). To further characterize the mutant phenotype, concentrated culture supernatant from RFB137 strain were analyzed by RP-HPLC and compared to that of the parental strain. As shown in Figure 2, the 21.5 min peak corresponding to amylolysin is lacking for RFB137 sample confirming that identified locus is involved in amylolysin biosynthesis. 

### Structural characterization

**Figure 3:**
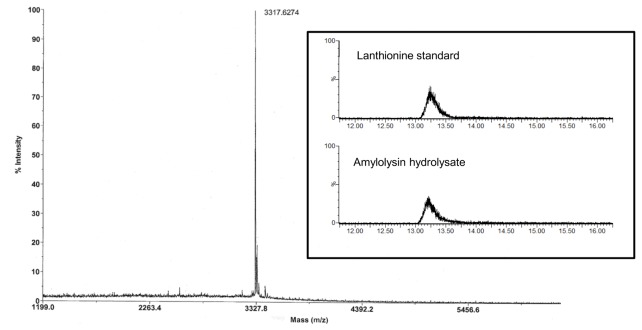
MALDI-TOF MS and LC-MS analysis of amylolysin. Mass spectrum of purified amylolysin sample was recorded in negative mode. Insert: LC-MS chromatograms of commercial lanthionine standard and amylolysin hydrolysate. Intensity (%, Y-scale) was recorded by setting the SQD mass analyzer on the specific mass of lanthionine (315 Da).

The presence of lanthionine, the structural characteristic trait of lantibiotics, was evidenced by LC-MS analysis. Amylolysin was first hydrolyzed in acidic conditions and OPA derivatized prior analysis (Figure **3** **insert**). In the resulting chromatogram, compounds eluted at retention time of 13.2 min in amylolysin hydrolysate and lanthionine standard samples, point out the presence of lanthionine [27]. Moreover, m/z signals at 315 deduced from the MS spectra confirm that amylolysin is a lantibiotic senso *stricto* (Figure **3** **, insert**). Similar results were obtained with the well-characterized lantibiotic nisin (data not shown). Matrix-assisted laser desorption ionization time of flight mass spectrometry analyses (MALDI-TOF) of purified amylolysin allowed the determination of its molecular mass (Figure **3**). Signal at m/z of 3317.6 obtained in negative mode of measurement highlighted a molecular mass of 3318.6 Da. The absence of this particular molecular mass in proteomic databases suggests that amylolysin could be a novel compound not described to date.

### Interaction with Lipid II

**Figure 4:**
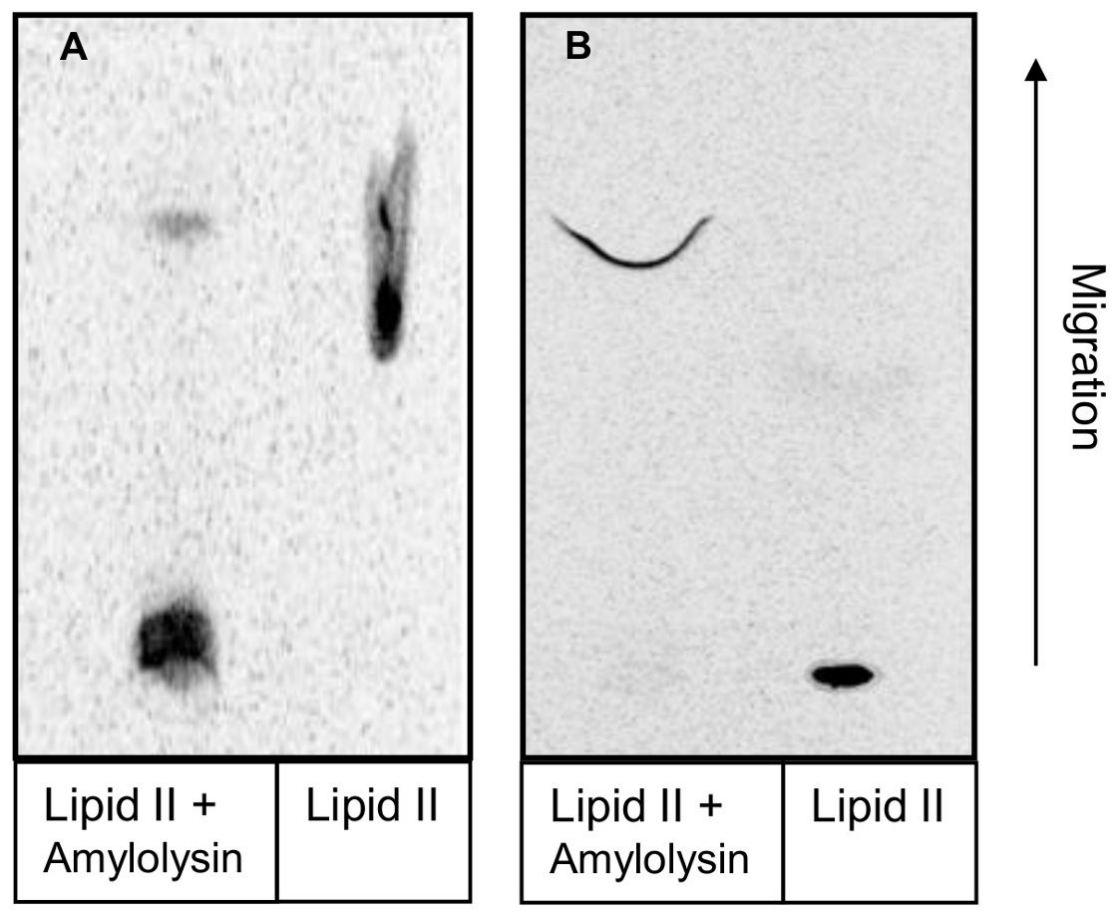
Interation with lipid II. (A) Direct interaction of amylolysin with lipid II. Both compounds were incubated in DMSO/1-octanol mixture (60/40 v/v) for 1 hour at room temperature before being subjected to TCL using a butanol/acetic acid/pyridine/water mixture (15/3/12/10, v/v/v/v) as mobile phase. (B) Inhibition of the transglycosylation reaction. Amylolysin, lipid II and glycosyltransferase were incubated for one hour before being subjected to TLC using a mixture of methanol/chloroform/ammoniac/water (88/48/10/1, v/v/v/v) as mobile phase. [^14^C] lipid II was detected with a Molecular Imager FX system. Direction of solvent migration during TLC is indicated by the arrow.

*In silico* analysis of AmlA highlighted the C^15^TLTWEC^21^ sequence as a putative interaction site between amylolysin and the lipid II. In order to confirm this hypothesis, two experiments were designed. In the first one, a direct amylolysin-lipid II interaction was characterized while in the second the ability of amylolysin to inhibit the transglycosylation reaction involved in peptidoglycan synthesis was investigated [28]. In both experiments, interactions were analyzed by thin layer chromatography based on the utilization of [^14^C] lipid II. As shown in Figure 4A, the delay of migration of the amylolysin-lipid II complex compared to that of free lipid II traduced the direct interaction between the two partners. By contrast, inhibition of the transglycosylation reaction resulted in a not polymerized lipid II that migrates compared to polymerized form remaining at the origin (Figure **4B**).

## Discussion

 The majority of the bacteriocins described to date are from lactic bacteria with nisin, used commercially in many countries as preservative in food products [29], being the best characterized compound. However, its sensitivity to proteases, its low solubility above pH 6 and emergence of nisin resistant strains, point out the need for alternative producer organisms [30]. In regards to their high level of protease production, *Bacillus* sp. has been considered as interesting alternative source for antimicrobial peptides with increased protease tolerance. In our laboratory, *B. amyloliquefaciens* GA1 has been characterized for its ability to produce antimicrobial metabolites [18]. Beside polyketides (PKs) and non-ribosomally synthesized peptides (NRPs), GA1 was found to produce amylolysin, a bacteriocin that is less sensitive to meat proteases than nisin [17].

 The prerequisites for the molecular characterization of amylolysin was to scale-up and to adapt the production process previously reported [17]. In addition, a GAI derivative RFB136 mutant, unable to produce NRPs and PKs, was used to prevent contaminations by other antimicrobial compounds during the purification process. 

The thermo-stability and pH tolerance together with the structural characterization by LC-MS demonstrated that amylolysin belongs to the class I bacteriocin family. Moreover, the presence of one unique AmlM modification enzyme and an AmlT transport protein with a peptidase-like associated domain suggests that amylolysin belongs to the type-B lantibiotics. The absence of significant similarity between AmlA and bacteriocins reported in BACTIBASE and Antimicrobial Peptide Database, points out that amylolysin corresponds to a novel lantibiotic not described to date. Reports on type-B lantibiotic from *B. amyloliquefaciens* are scarce. So far, a gene cluster for lantibiotic synthesis has been reported in *B. amyloliquefaciens* subsp. *plantarum* strain YAU B9601-Y2 [31]. Beside this, mersacidin production has been reported recently in *B. amyloliquefaciens* subsp. *plantarum* B6901-Y2 and *B. amyloliquefaciens* HIL Y-85,54728 [7,15,16]. The biosynthetic cluster consists in a structural gene *mrsA*, together with gene *mrsM*, involved in posttranslational modifications of the mersacidin prepeptide; *mrsT*, coding for a transporter with associated protease domain. The immunity genes *mrsF*, *mrsE* and *mrsG* and the regulatory genes *mrsR1*, *mrsR2* and *mrsK2*, respectively, are also present. The two-component regulatory system MrsR2/MrsK2 is mainly involved in immunity and self-induction of mersacidin biosynthesis [32] whereas Mrs1 was found essential for mersacidin production [33]. The presence of genes involved in mersacidin self-protection has been also detected in the genome of *B. amyloliquefaciens* FZB42 [34]. Despite that strain FZB42 is unable to produce mersacidin, it has been used recently as a host cell for the transfer of the mersacidin biosynthesis genes from *B. amyloliquefaciens* HIL Y-85,54728 [15].

A close inspection of *amlA* sequence showed that it codes for a 60 residues prepeptide with a specific G^26^GGG^29^ motif. Type-B lantibiotics are known to possess leader sequences ranging from 15 to 40 amino acids that are processed within G(G/A/S)\XX motif. Thus, the G^26^GGG^29^ sequence found in AmlA is the best candidate for processing site of the signal peptide. However, the presence of four glycine residues allows three possibilities for this processing (i.e. after glycine at position 27, 28 and 29, respectively). MALDI-TOF measurement has highlighted a mature AmlA peptide of 3318 Da that is consistent with a cleavage by AmlM after G^29^. Thioether bridging patterns have been determined for several type-B lantibiotics including lacticin 3147 [35] and haloduracin [21]. Off these, the conserved CTxTxEC motif was found involved in the formation of one Lan residue and two MeLan creating the three-dimensional structure responsible for the interaction with lipid II. Based on sequence alignment with α-peptide of lacticin 3147 and haloduracin, it is likely that these posttranslational modifications in amylolysin occur between amino acid 36-44, 46-49 and 47-52 of the mature peptide, respectively (Figure **1C**). In addition, AmlA is characterized by a mass that is consistent, with two possible Dhb residues at amino acid position 55 and 57. Structurally, amylolysin seems more related to α-peptide of two-component lantibiotics compared to other lantibiotics described among the *Bacillus* genus such as mersacidin or subtilin.

 For most of the lantibiotics from *Bacillus* sp. described so far, immunity is conferred by LanI and/or LanFEG proteins, with exception of entianin and lacticin 3147 whose immunity seems to be supported by EntG/EntI and LtnEF/LtnI proteins, respectively [10,36] Moreover, in gallidermin producer strain, an additional LanH protein, which serves as an ancillary protein for the LanFE protein, has been described [37]. Mechanistically, LanI peptides are thought to function by lantibiotic interception or target shielding [12,38] whereas LanFE(G) proteins form ABC transporter of two or three subunits with LanF being the ATP-binding domain [39]. In lacticin 3147 producer strains, it has been shown that LtnE and LtnF play an important role in immunity mechanism even at a greater degree than LtnI. From this viewpoint, amylolysin gene cluster is also atypical. Only two genes, namely AmlF and AmlE, have been detected in GA1 strain suggesting that immunity to amylolysin is ensured by a two-component ABC transporter. The immunity mechanism is therefore somewhat different from that found in *B. amyloliquefaciens* for mersacidin self-protection [15,16].

This first characterization of amylolysin highlights some divergences with the one-component type-B lantibiotic usually produced by *Bacillus* sp. However, structural as well as mechanistic data are still lacking to deeply characterize this promising lantibiotics. The fact that amylolysin interacts with lipid II, is certainly a first element to explain its biological mode of action. Experiments are in progress to get those lacking information.

## Materials and Methods

### Bacterial strains, culture media and general genetic techniques

*Bacillus amyloliquefaciens* GA1 was used for amylolysin production [17]. The different bacterial and hyphal isolates used as indicator strains are listed in table 1. *Escherichia coli* DH5α (Promega, Madisson, WI, USA) was used for transformation and amplification of recombinant plasmid DNA according standard procedures [40]. *B. amyloliquefaciens* GA1 was transformed as described elsewhere [41]. Luria-Bertani (LB), brain-heart (BH), de Man-Rogosa-Sharpe (MRS), and YPD media were of commercial origin (Becton-Dickinson, Le pont de Chaix, France). Microbial growth was monitored by optical density measurement at 600 nm (OD_600_). Genomic DNA was purified using the Wizard genomic purification kit (Promega). PCR amplifications were performed with Fideli Taq polymerase (USB Corporation, Cleveland, OH, USA) and amplified fragments were purified with the Qiagen purification kit (Hilden, Germany). PCR fragments were cloned into pGEM-T-easy vector prior sequencing at GIGA Genomics Facilities (University of Liège, Liège, Belgium). Primers used for PCR amplifications, plasmids and bacterial strains are listed in Table **S1**. For similarity searches, the FastA program [42] was used to scan UniProtKB/Swiss-Prot and UniProtKB/TrEMBL databases. Sequence alignments were generated using ClustalW (http://www.ebi.ac.uk/Tools/msa/clustalw2/) and manually adjusted in Genedoc (http://www.nrbsc.org/gfx/genedoc/) to correct obvious mispairings. Predictions terminators were performed using ARNold program [43] whereas BPROM program was used for prediction of putative promoter elements (http://linux1.softberry.com/all.htm). Blasts analysis were performed at the NCBI website (http://blast.ncbi.nlm.nih.gov/Blast.cgi).

### Amylolysin production, purification and quantification

Large-scale production of amylolysin was performed in a 80-liters Bioflo 5000 bioreactor (New Brunswick) in a working volume of 60 L of LB medium with strain RFB136 (a derivative of strain GA1 unable to produce lipopeptides, see Text **S1**). Cultures were conducted at 37 °C for 10 h at a stirring speed of 200 rpm and an aeration flow of 1 VVM (volume of air per volume of medium per minute). Cell-free supernatant was obtained by cross flow filtration using a hollow fiber cartridge (0.45 µm, 8400 cm^2^, GE Healthcare) according to the manufacturer’s recommendations. Amylolysin was purified in a three steps procedure. Firstly, the antimicrobial peptide was extracted from the culture supernatant using an Amberlite XAD-16 resin (Sigma-Aldrich) and concentrated by rotary evaporation. Amylolysin was subsequently purified in two steps of reverse-phase RP-HPLC using a VP Nucleosil column (250 x 10 mm, 7 μm packing, Macherey-Nagel) and a Nucleodur C18 column (50 x 4.6 mm, 5 μm packing, Macherey-Nagel) at a flow rate of 2.5 and 1 ml min^-1^ of an acetonitrile/H_2_O mixture (41/59, v/v), respectively. Amylolysin identification and quantification were performed by RP-HPLC as described previously [17].

### Effect of enzyme, pH and temperature on amylolysin activity

To evaluate sensitivity to proteases, purified amylolysin samples were quantified by RP-HPLC after 24h of incubation at 37°C with proteinase K and pronase (10 µg/ml, Sigma-Aldrich). Heat stability was evaluated after incubation for 60 min at various temperatures (25, 55, 65, 75 and 100 °C) whereas pH sensitivity was evaluated after a 1h-incubation in 200 mM HCl/KCl buffer (pH 2-3), acetate buffer (pH 4-6) and Tris buffer (pH 7-9). After those treatments, the remaining amylolysin activity was determined using the agar diffusion assays as described elsewhere, using *Micrococcus luteus* ATCC 9341 as an indicator strain.

### Minimal inhibitory concentration determination

Minimal inhibitory concentration (MIC) was determined for an array of bacterial and yeast strains by a microdilution method following the Clinical and Laboratory Standard Institute (CLSI) recommended procedure [44]. Briefly, the different indicator strains were aerobically grown in 24-wells culture plates in the presence of purified amylolysin at concentration ranging from 0.1 to 2.8 µM in the appropriate medium and temperature conditions as stipulated in table 1. Each well was seeded at an OD_600_ of 0.1. After 24 h of incubation, cell growth was estimated based on OD_600_ measurements. The MIC values were defined as the lowest amylolysin concentration that is able to inhibit cell growth. Each experiment was performed in triplicate.

### Characterization of amylolysin biosynthesis genes

Identification of the genomic locus involved in amylolysin biosynthesis was performed by inverse polymerase chain reaction (IPCR) [19]. Briefly, 5 µg of purified genomic DNA of *B. amyloliquefaciens* GA1 were first digested with restriction enzyme *Ps*tI (1U/µg for 2h). The digested DNA was then purified before being self-ligated using T4 DNA ligase (Promega). PCR amplification was then performed using primer pair RFO142/RFO143 and the self-ligation mixture as a template with an elongation time of 10 min. The resulting PCR fragment was then cloned into pGEM-T-easy vector and sequenced by primer walking. The resulting sequence was then used for Blast search for further characterization of the amylolysin locus.

### Disruption of *amlA*

Disruption of *amlA* was performed as described previously [45]. Briefly, the 407 and 523 bp P and T fragment consisting of part of the 3’ and 5’ *amlA* ORF were PCR-amplified using primer pairs P2B4sens/P2B4rev and T2B4sens/T2B4rev, respectively, and *B. amyloliquefaciens* GA1 genomic DNA as a template. Primers P2B4rev and T2B4sens contain the rare meganuclease I-*SceI* recognition sequence. P-ISceI and ISceI-T fragments were then pooled and used as a template for amplification of the P-ISceI-T cassette with primers P2B4sens and P2B4rev. The resulting fragment was then cloned into pGEM-T easy vector to generate RFPA1. The 1.6 kb fragment encoding a kanamycin resistance gene was rescued from RFP104 [41] by *ISceI* digestion and subcloned into RFPA1 at the corresponding restriction site to yield RFPA2. The *amlA* disruption cassette was finally obtained by PCR amplification with primers pair P2B4sens/T2B4rev and RFPA2 as a template and used, after purification, to transform *B. amyloliquefaciens* RFB136. Transformants were selected on LB-kanamycin plates (10 µg/ml). Integration by double-crossing event in the RFB136 strain was verified by analytical PCR using primer pair P2B4sens/T2B4rev. The *amlA*-disrupted strain was denominated RFB137.

### Mass spectrometry

The molecular mass of the purified amylolysin was determined by matrix-assisted laser desorption ionization-time of flight (MALDI-TOF) mass spectrometry using a 4700 Proteomics analyzer (Applied Biosystem). Purified amylolysin was mixed with an equal volume of α-cyanohydroxycinnamic acid solution (10 mg/ml) and spotted onto the MALDI plate. The analyzer was used at an acceleration voltage of 20 kV. Samples were measured in the reﬂectron mode. Mass spectral databases and proteomic tools were MassBank (http://www.massbank.jp) and Prospector (http://prospector.ucsf.edu/).

The presence of the modified amino acid lanthionine in amylolysin was demonstrated by RP-HPLC coupled with single quad mass spectrometer (HPLC Waters Alliance 2695/diode array detector, coupled with Waters SQD mass analyzer) on a X-terra MS column (150 x 2.1 mm, 3.5 μm packing, Waters) after peptide hydrolysis (HCl 6 M, for 4 h à 145°C) and derivatization with o-phthaldialdehyde (OPA) (Agilent Technology) as described elsewhere [46]. Elution was performed at a constant ﬂow rate of 0.25 ml min^-1^ and at 40°C, with a gradient of acetonitrile (solvent B) in water acidiﬁed with 0.1% formic acid (solvent A) as follows: 0-2 min, 0% B; 2-7 min, 0 to 10% B; 7-17 min, 10 to 15% B; 17-19 min, 15 to 95% B). Compounds were ﬁrst identiﬁed on the basis of their retention times compared with a commercial lanthionine standard (Sigma-Aldrich). The identity of lanthionine was subsequently conﬁrmed on the basis of the masses detected in the SQD by setting electrospray ionization (positive ion) conditions in the MS as source temperature, 130°C; desolvation temperature, 250°C; nitrogen ﬂow, 500 l h-1; cone voltage, 50 V. 

### Interaction with Lipid II

Radiolabelled UDP-MurNac-pentapeptide, thereafter [^14^C] lipid II, was prepared *in vitro* using *E. coli* K12 purified membrane from UDP-GlcNac and UDP-MurNAc-L-Ala-D-Glu-meso-DAP-D-[^14^C]Ala-D-[^14^C]Ala and subsequently purified as previously described [28]. Inhibition of glycosyl transferase reaction was performed as described elsewhere [47] with some modifications. Briefly, [^14^C] lipid II (2.5µM; 0.125µCi nmol^-1^), amylolysin (0.2 µM), His-tag PBP1bγ from *E. coli* (15 nM) were incubated in 25mM Tris-HCl (pH7,5), 0,5 % decyl PEG, 10 mM MgCl_2_, 12% 1-octanol, 25 % DMSO for 1 hour at 30 °C. The reaction products were separated by thin layer chromatography (TLC) on silica plate (SilG, 250 µM thickness, Macherey-Nagel, Duren, Germany) using a mixture of methanol/chloroform/ammoniac/water (88/48/10/1, v/v/v/v) as mobile phase. For interaction experiment, [^14^C] lipid II (2.5 µM; 0.125µCinmol^-1^) and amylolysin (0.2 µM) were incubated in DMSO/1-octanol mixture (60/40 v/v) for 1 hour at room temperature before being subjected to TCL using a butanol/acetic acid/pyridine/water mixture (15/3/12/10, v/v/v/v) as mobile phase. The radioactive compounds were detected and analyzed with a Molecular Imager FX system (Biorad Laboratories).

## Supporting Information

Figure S1 **Sequence alignment of the N-terminus of type B lantibiotic modification enzymes.**
(DOCX)

Table S1 **Strains and plasmids used in this study.**
(DOCX)

Text S1 **Construction of strain RFB136.**
(DOCX)

## References

[1] Swaminathan B, Gerner-Smidt P (2007) The epidemiology of human listeriosis. Microbes Infect 9: 1236-1243. doi:10.1016/j.micinf.2007.05.011. PubMed: 17720602. 17720602

[2] Piper C, Cotter PD, Ross RP, Hill C (2009) Discovery of medically significant lantibiotics. Curr Drug Discov Technol 6: 1-18. doi:10.2174/157016309787581075. PubMed: 19275538. 19275538

[3] Schnell N, Entian KD, Schneider U, Götz F, Zähner H et al. (1988) Prepeptide sequence of epidermin, a ribosomally synthesized antibiotic with four sulphide-rings. Nature 333: 276-278. doi:10.1038/333276a0. PubMed: 2835685. 2835685

[4] Kaletta C, Entian KD (1989) Nisin, a peptide antibiotic: cloning and sequencing of the nisA gene and posttranslational processing of its peptide product. J Bacteriol 171: 1597-1601. PubMed: 2493449. 2493449 10.1128/jb.171.3.1597-1601.1989 PMC209786

[5] Appleyard AN, Choi S, Read DM, Lightfoot A, Boakes S et al. (2009) Dissecting structural and functional diversity of the lantibiotic mersacidin. Chem. Biol 16: 490-498. 10.1016/j.chembiol.2009.03.011 PMC2706954 19477413

[6] Altena K, Guder A, Cramer C, Bierbaum G (2000) Biosynthesis of the lantibiotic mersacidin: organization of a type B lantibiotic gene cluster. Appl Environ Microbiol 66: 2565-2571. doi:10.1128/AEM.66.6.2565-2571.2000. PubMed: 10831439. 10831439 PMC110582

[7] Brötz H, Bierbaum G, Markus A, Molitor E, Sahl HG (1995) Mode of action of the lantibiotic mersacidin: inhibition of peptidoglycan biosynthesis via a novel mechanism? Antimicrob Agents Chemother 39: 714-719. doi:10.1128/AAC.39.3.714. PubMed: 7793878. 7793878 PMC162610

[8] Sass P, Jansen A, Szekat C, Sass V, Sahl HG et al. (2008) The lantibiotic mersacidin is a strong inducer of the cell wall stress response of Staphylococcus aureus. BMC Microbiol 8: 186. doi:10.1186/1471-2180-8-186. PubMed: 18947397. 18947397 PMC2592248

[9] Kordel M, Schüller F, Sahl HG (1989) Interaction of the pore forming-peptide antibiotics Pep 5, nisin and subtilin with non-energized liposomes. FEBS Lett 244: 99-102. doi:10.1016/0014-5793(89)81171-8. PubMed: 2924913. 2924913

[10] Suda S, Cotter PD, Hill C, Ross RP (2012) Lacticin 3147--biosynthesis, molecular analysis, immunity, bioengineering and applications. Curr Protein Pept Sci 13: 193-204. doi:10.2174/138920312800785021. PubMed: 21827422. 21827422

[11] Håvarstein LS, Diep DB, Nes IF (1995) A family of bacteriocin ABC transporters carry out proteolytic processing of their substrates concomitant with export. Mol Microbiol 16: 229-240. doi:10.1111/j.1365-2958.1995.tb02295.x. PubMed: 7565085. 7565085

[12] Stein T, Heinzmann S, Düsterhus S, Borchert S, Entian KD (2005) Expression and functional analysis of the subtilin immunity genes spaIFEG in the subtilin-sensitive host Bacillus subtilis MO1099. J Bacteriol 187: 822-828. doi:10.1128/JB.187.3.822-828.2005. PubMed: 15659659. 15659659 PMC545732

[13] Klein C, Kaletta C, Entian KD (1993) Biosynthesis of the lantibiotic subtilin is regulated by a histidine kinase/response regulator system. Appl Environ Microbiol 59: 296-303. PubMed: 8439156. 8439156 10.1128/aem.59.1.296-303.1993 PMC202094

[14] Delves-Broughton J, Blackburn P, Evans RJ, Hugenholtz J (1996) Applications of the bacteriocin, nisin. Antonie Van Leeuwenhoek 69: 193-202. doi:10.1007/BF00399424. PubMed: 8775979. 8775979

[15] Herzner AM, Dischinger J, Szekat C, Josten M, Schmitz S et al. (2011) Expression of the lantibiotic mersacidin in Bacillus amyloliquefaciens FZB42. PLOS ONE 6: e22389. doi:10.1371/journal.pone.0022389. PubMed: 21811596. 21811596 PMC3141056

[16] He P, Hao K, Blom J, Rückert C, Vater J et al. (2012) Genome sequence of the plant growth promoting strain Bacillus amyloliquefaciens subsp. plantarum B9601-Y2 and expression of mersacidin and other secondary metabolites. J Biotechnol 164: 281-291. doi:10.1016/j.jbiotec.2012.12.014. PubMed: 23357245. 23357245

[17] Halimi B, Dortu C, Arguelles Arias A, Thonart P, Joris B et al. (2010) Antilisterial Activity on Poultry Meat of Amylolysin, a Bacteriocin from *Bacillus* *amyloliquefaciens* GA1. Probiotics and Antimicrob Prot 2: 120-125. 10.1007/s12602-010-9040-9 26781121

[18] Arguelles-Arias A, Ongena M, Halimi B, Lara Y, Brans A et al. (2009) Bacillus amyloliquefaciens GA1 as a source of potent antibiotics and other secondary metabolites for biocontrol of plant pathogens. Microb Cell Fact 8: 63. doi:10.1186/1475-2859-8-63. PubMed: 19941639. 19941639 PMC2787494

[19] Ochman H, Gerber AS, Hartl DL (1988) Genetic applications of an inverse polymerase chain reaction. Genetics 120: 621-623. PubMed: 2852134. 2852134 10.1093/genetics/120.3.621 PMC1203539

[20] Cotter PD, Deegan LH, Lawton EM, Draper LA, O'Connor PM et al. (2006) Complete alanine scanning of the two-component lantibiotic lacticin 3147: generating a blueprint for rational drug design. Mol Microbiol 62: 735-747. doi:10.1111/j.1365-2958.2006.05398.x. PubMed: 17076667. 17076667

[21] Cooper LE, McClerren AL, Chary A, van der Donk WA (2008) Structure-activity relationship studies of the two-component lantibiotic haloduracin. Chem Biol 15: 1035-1045. doi:10.1016/j.chembiol.2008.07.020. PubMed: 18940665. 18940665 PMC2633096

[22] Siezen RJ, Kuipers OP, de Vos WM (1996) Comparison of lantibiotic gene clusters and encoded proteins. Antonie Van Leeuwenhoek 69: 171-184. doi:10.1007/BF00399422. PubMed: 8775977. 8775977

[23] Sanders DA, Gillece-Castro BL, Stock AM, Burlingame AL, Koshland DE Jr. (1989) Identification of the site of phosphorylation of the chemotaxis response regulator protein, CheY. J Biol Chem 264: 21770-21778. PubMed: 2689446. 2689446

[24] Engelke G, Gutowski-Eckel Z, Kiesau P, Siegers K, Hammelmann M et al. (1994) Regulation of nisin biosynthesis and immunity in Lactococcus lactis 6F3. Appl Environ Microbiol 60: 814-825. PubMed: 8161176. 8161176 10.1128/aem.60.3.814-825.1994 PMC201397

[25] Nishie M, Sasaki M, Nagao J, Zendo T, Nakayama J et al. (2011) Lantibiotic transporter requires cooperative functioning of the peptidase domain and the ATP binding domain. J Biol Chem 286: 11163-11169. doi:10.1074/jbc.M110.212704. PubMed: 21303905. 21303905 PMC3064170

[26] Barrett AJ, Rawlings ND, O'Brien EA (2004) Handbook of Proteolytic Enzymes. Academic Press p. 2.

[27] Sahl HG, Hahn C, Brandis H (1985) Interaction of the staphylococcin-like peptide Pep 5 with cell walls and isolated cell wall components of Gram-positive bacteria. Zentralbl Bakteriol Mikrobiol Hyg A 260: 197-205 10.1016/s0176-6724(85)80115-2 4082823

[28] van Heijenoort Y, Gómez M, Derrien M, Ayala J, van Heijenoort J (1992) Membrane intermediates in the peptidoglycan metabolism of Escherichia coli: possible roles of PBP 1b and PBP 3. J Bacteriol 174: 3549-3557. PubMed: 1592809. 1592809 10.1128/jb.174.11.3549-3557.1992 PMC206040

[29] Ross RP, Morgan S, Hill C (2002) Preservation and fermentation: past, present and future. Int J Food Microbiol 79: 3-16. doi:10.1016/S0168-1605(02)00174-5. PubMed: 12382680. 12382680

[30] Dischinger J, Josten M, Szekat C, Sahl HG, Bierbaum G (2009) Production of the novel two-peptide lantibiotic lichenicidin by Bacillus licheniformis DSM 13. PLOS ONE 4: e6788. doi:10.1371/journal.pone.0006788. PubMed: 19707558. 19707558 PMC2727956

[31] Hao K, He P, Blom J, Rueckert C, Mao Z et al. (2012) The genome of plant growth-promoting Bacillus amyloliquefaciens subsp. plantarum strain YAU B9601-Y2 contains a gene cluster for mersacidin synthesis. J Bacteriol 194: 3264-3265. doi:10.1128/JB.00545-12. PubMed: 22628498. 22628498 PMC3370840

[32] Schmitz S, Hoffmann A, Szekat C, Rudd B, Bierbaum G (2006) The lantibiotic mersacidin is an autoinducing peptide. Appl Environ Microbiol 72: 7270-7277. doi:10.1128/AEM.00723-06. PubMed: 16980420. 16980420 PMC1636175

[33] Guder A, Schmitter T, Wiedemann I, Sahl HG, Bierbaum G (2002) Role of the single regulator MrsR1 and the two-component system MrsR2/K2 in the regulation of mersacidin production and immunity. Appl Environ Microbiol 68: 106-113. doi:10.1128/AEM.68.1.106-113.2002. PubMed: 11772616. 11772616 PMC126572

[34] Chen XH, Koumoutsi A, Scholz R, Eisenreich A, Schneider K et al. (2007) Comparative analysis of the complete genome sequence of the plant growth-promoting bacterium Bacillus amyloliquefaciens FZB42. Nat Biotechnol 25: 1007-1014. doi:10.1038/nbt1325. PubMed: 17704766. 17704766

[35] Martin NI, Sprules T, Carpenter MR, Cotter PD, Hill C et al. (2004) Structural characterization of lacticin 3147, a two-peptide lantibiotic with synergistic activity. Biochemistry 43: 3049-3056. doi:10.1021/bi0362065. PubMed: 15023056. 15023056

[36] Fuchs SW, Jaskolla TW, Bochmann S, Kötter P, Wichelhaus T et al. (2011) Entianin, a novel subtilin-like lantibiotic from Bacillus subtilis subsp. spizizenii DSM 15029T with high antimicrobial activity. Appl Environ Microbiol 77: 1698-1707. doi:10.1128/AEM.01962-10. PubMed: 21239550. 21239550 PMC3067280

[37] Otto M, Peschel A, Götz F (1998) Producer self-protection against the lantibiotic epidermin by the ABC transporter EpiFEG of Staphylococcus epidermidis Tu3298. FEMS Microbiol Lett 166: 203-211. doi:10.1016/S0378-1097(98)00333-4. PubMed: 9770275. 9770275

[38] Hoffmann A, Schneider T, Pag U, Sahl HG (2004) Localization and functional analysis of PepI, the immunity peptide of Pep5-producing Staphylococcus epidermidis strain 5. Appl Environ Microbiol 70: 3263-3271. doi:10.1128/AEM.70.6.3263-3271.2004. PubMed: 15184120. 15184120 PMC427782

[39] Draper LA, Grainger K, Deegan LH, Cotter PD, Hill C et al. (2009) Cross-immunity and immune mimicry as mechanisms of resistance to the lantibiotic lacticin 3147. Mol Microbiol 71: 1043-1054. doi:10.1111/j.1365-2958.2008.06590.x. PubMed: 19183281. 19183281

[40] Shambrook J, Fritch EF, Maniatis M (1989) Molecular Cloning: A laboratory Manual 2nd Edition. Cold Spring Harbar Lab Press, New York.

[41] Fickers P, Leclère V, Guez JS, Béchet M, Coucheney F et al. (2008) Temperature dependence of mycosubtilin homologue production in Bacillus subtilis ATCC6633. Res Microbiol 159: 449-457. doi:10.1016/j.resmic.2008.05.004. PubMed: 18656330. 18656330

[42] Pearson WR, Lipman DJ (1988) Improved tools for biological sequence comparison. Proc Natl Acad Sci U S A 85: 2444-2448. doi:10.1073/pnas.85.8.2444. PubMed: 3162770. 3162770 PMC280013

[43] Naville M, Ghuillot-Gaudeffroy A, Marchais A, Gautheret D (2011) ARNold: a web tool for the prediction of Rho-independent transcription terminators. RNA Biol 8: 11-13. doi:10.4161/rna.8.1.13346. PubMed: 21282983. 21282983

[44] CaLS (2009) Methods for dilution antimicrobial susceptibility tests for bacteria that growth aerobically; approved standard. CLSI M7: A8.

[45] Fickers P, Le Dall MT, Gaillardin C, Thonart P, Nicaud JM (2003) New disruption cassettes for rapid gene disruption and marker rescue in the yeast Yarrowia lipolytica. J Microbiol Methods 55: 727-737. doi:10.1016/j.mimet.2003.07.003. PubMed: 14607415. 14607415

[46] Sahl HG, Grossgarten M, Widger WR, Cramer WA, Brandis H (1985) Structural similarities of the staphylococcin-like peptide Pep-5 to the peptide antibiotic nisin. Antimicrob Agents Chemother 27: 836-840. doi:10.1128/AAC.27.5.836. PubMed: 4015073. 4015073 PMC180162

[47] Terrak M, Ghosh TK, van Heijenoort J, Van Beeumen J, Lampilas M et al. (1999) The catalytic, glycosyl transferase and acyl transferase modules of the cell wall peptidoglycan-polymerizing penicillin-binding protein 1b of Escherichia coli. Mol Microbiol 34: 350-364. doi:10.1046/j.1365-2958.1999.01612.x. PubMed: 10564478. 10564478

