# Supplementary material for: Characterization of Amylolysin, a Novel Lantibiotic from Bacillus amyloliquefaciens GA1
Source: PLoS One. 2013 Dec 9;8(12):e83037. doi: 10.1371/journal.pone.0083037 (PMC3857288; doi:10.1371/journal.pone.0083037)
Supplement: Figure S1 — Sequence alignment of the N-terminus of type B lantibiotic modification enzymes. (DOCX) [file pone.0083037.s002.docx]

**Figure S1: Sequence alignment of the N-terminus of type B lantibiotic modification enzymes.**

N1 N2 N3 N4

AmlM (164)-LA**G**ETPKEK-(72)-GMG**D**S**H**SDGRK-(10)-LI**YKP**R-(56)-FYQRT**G**AQIGLLYALKAV**D**F**H**SE**N**L**I**ANGSY**P**VLI**D**L**E**SL-

LasM ( 73)-ML**G**NNSSER-(70)-GKG**D**T**H**SGKSV-(15)-LL**YKP**R-(53)-YYYRS**G**CLLGLFWILGTT**D**I**H**SE**N**I**I**TNAGY**P**IVI**D**I**E**TL-

MutM (56)-LI**G**LTSEEK-(70)-VVG**D**M**H**NELAV-( 9)-LF**YKP**H-(53)-YFSRM**G**GLIAIAYSLNMT**D**L**H**FE**N**I**I**SDGEY**P**VIL**D**M**E**TI-

MrsM (180)-LR**G**ETSEER-(73)–GVS**D**T**H**NKGKT-(10)–IV**YKP**R-(54)-FYWRI**G**SYLAILYAMNAV**D**F**H**MQ**N**L**I**ADGEY**P**ILV**D**L**E**SL-

CylM (169)-LK**G**NDSSKR-(70)–SQG**D**S**H**SRGKT-(11)–IV**YKP**K-(52)-YYERY**G**KLIGIAFLFNVT**D**L**H**YE**N**I**I**AHGEY**P**VII**D**N**E**TF-

LctM (56)-LM**G**NTPEER-(70)–IKG**D**L**H**NGKAV-( 9)-LI**YKP**K-(62)-YYRKI**G**VLLSVAYTLNLT**D**L**H**FE**N**V**I**SQGEN**P**CII**D**L**E**TM-

: * ..:: .* *. . :.*** :: : * :.: : :. .*:* :*:*:.. * ::* *::

N5 N6

AmlM (24)-**S**VRALGLL**P**-(106)–**R**FISKP**T**IK**Y**SALLELSFH**P**-(364)

LasM (19)-**S**VLSSCLL**P**-( 97)-**R**QLLRD**T**QV**Y**ADFVHALQL**P**-(503)

MutM (25)-**S**VLNTGLL**P**-(103)-**R**VIFRQ**T**AH**Y**SLMLEVLNS**P**-(487)

MrsM (22)-**S**VLRIGLL**P**-(102)–**R**QILRG**T**SR**Y**ANLLKISLH**P**-(523)

CylM (23)-**S**IMVTGLV**P**-(105)–**R**NVIRP**T**QR**Y**ADMLEFSYH**P**-(468)

LctM (24)-**S**VVSTGML**P**-(103)–**R**ILFRN**T**ME**Y**SVLLNAAKS**P**-(503)

*:: ::* * : : * *: ::. *
