# Supplementary material for: Characterization of Amylolysin, a Novel Lantibiotic from Bacillus amyloliquefaciens GA1
Source: PLoS One. 2013 Dec 9;8(12):e83037. doi: 10.1371/journal.pone.0083037 (PMC3857288; doi:10.1371/journal.pone.0083037)
Supplement: Table S1 — Strains and plasmids used in this study. (DOCX) [file pone.0083037.s003.docx]

**Table S1: Strains and plasmids used in this study**

| **Table S1**. Strains and plasmids used in this study | | |
| --- | --- | --- |
| Strain (host strain) | Plasmid, genotype or sequence (5’→ 3’) | Reference, source or restriction site |
| ***E. coli* strains** |  |  |
| DH5α | Φ80d*lac*ZΔm15, *rec*A1, *end*A1, *gyr*A96, *thi*-1, *hsd*R17 (r_k_-, m_k_+), *sup*E44, *rel*A1, *deo*R, Δ(*lac*ZYA*-arg*F)U169 | Promega |
| RFE104 (DH5α) | RFP104, ISceI-kan-ISceI fragment | [1] |
| RFEA1 (DH5α) | RFPA1, 0.9 kb *amyA* PT cassette | This work |
| RFEA1 (DH5α) | RFPA2, 1.6 kb kan fragment into amyA PT cassette | This work |
| *B. amyloliquefaciens* GA1 | wild-type strain | [2] |
| RFB136 | GA1 derivative, *sfp::spc* | This work |
| RFB137 | RFB136 derivative, *sfp::spc, amlA::kan,* | This work |
| **Primers** |  |  |
| RFO142 | GAATGAGAAAGACTTACTAAGCTCA | - |
| RFO143 | GAATGAGAATCACCCATCCC | - |
| P2B4sens | TGAGCTTAGTAAGTCTTTCTCATTC | - |
| P2B4rev | CATTACCCTGTTATCCCTAACTATGTCGAAATTGATACAGC | I-*SceI* |
| T2B4sens | CTAGGGATAACAGGGTAATTGCTTTCCCTTTCCGACCTA | I-*SceI* |
| T2B4rev | GGGATGGGTGATTCTCATTC | - |
| Sfp1 | GGATGGTTTTGACAATCTTTTGCAG | - |
| Sfp2_SfiI | AATAGGCCTGTTTGGCCGCCTGCTTATGACTGAGCGAACG | *SfiI* |
| Sfp3_SfiI | ATGTGGCCAAAACGGCCCAGGAATACGGGAAGCCGTGC | *SfiI* |
| Sfp4 | GATGCAGGCGCACTGAAAAGG | - |
| SPCup_SfiI | ATGTggccaaacaggcccccccgggctgcagtagggataac | *SfiI* |
| SPCdw_SfiI | aataggccgttttggcccccccctcgagattaccctg | *SfiI* |
