# Supplementary material for: Characterization of Amylolysin, a Novel Lantibiotic from Bacillus amyloliquefaciens GA1
Source: PLoS One. 2013 Dec 9;8(12):e83037. doi: 10.1371/journal.pone.0083037 (PMC3857288; doi:10.1371/journal.pone.0083037)
Supplement: Text S1 — Construction of strain RFB136. (DOCX) [file pone.0083037.s004.docx]

**Text S1: Construction of strain RFB136**

Strain RFB136 was obtained from strain GA1 by disruption of the *sfp* gene. The disruption cassette was obtained by a ligation-mediated PCR method as previously described [[1](#_ENREF_1)]. Fragment of 200 and 300 bp consisting of part of the 3’ and 5’ of *sfp* ORF were PCR-amplified using primer pairs sfp1/sfp2_sfi and sfp3_sfi/Sfp4, respectively, and *B. amyloliquefaciens* GA1 genomic DNA as a template. Beside this, the spectinomycin resistance cassette (SPC) of RFP126 (Fickers et al, unpublished) was amplified with primer SPCup_SfiI and SPCdw_SfiI. PCR fragment (200 ng) were digested by *Sfi*I, purified and ligated with T4 DNA ligase. The 3’sfp-SPC-5’sfp was then purified from gel electrophoresis before being used as a template for PCR amplification using primer pair sfp1/sfp4. The sfp disruption cassette was then used to transform *B. amyloliquefaciens* GA1. Transformants were selected on LB-spectinomycin plates. Integration by double-crossing over event in the RFB136 resulting strain was verified by liquid chromatography-electrospray ionization-mass spectrometry as described elsewhere [[2](#_ENREF_2)].

1. Fickers P, Guez JS, Damblon C, Leclere V, Bechet M, et al. (2009) High-level biosynthesis of the anteiso-C(17) isoform of the antibiotic mycosubtilin in Bacillus subtilis and characterization of its candidacidal activity. Appl Environ Microbiol 75: 4636-4640.

2. Arguelles-Arias A, Ongena M, Halimi B, Lara Y, Brans A, et al. (2009) Bacillus amyloliquefaciens GA1 as a source of potent antibiotics and other secondary metabolites for biocontrol of plant pathogens. Microb Cell Fact 8: 63.
